# Cross-sectional analysis of follow-up chest MRI and chest CT scans in patients previously affected by COVID-19

**DOI:** 10.1007/s11547-021-01390-4

**Published:** 2021-07-12

**Authors:** Martina Pecoraro, Stefano Cipollari, Livia Marchitelli, Emanuele Messina, Maurizio Del Monte, Nicola Galea, Maria Rosa Ciardi, Marco Francone, Carlo Catalano, Valeria Panebianco

**Affiliations:** 1 Department of Radiological Sciences, Oncology and Pathology, Sapienza/Policlinico Umberto I, Viale Regina Elena 324, 00161 Rome, Italy; 2 Department of Public Health and Infectious Disease, Sapienza/Policlinico Umberto I, Viale Regina Elena 324, 00161 Rome, Italy; 3 Università degli Studi di Roma La Sapienza, Rome, Italy

**Keywords:** Chest computerized tomography, Chest magnetic resonance imaging, COVID-19, Pneumonia

## Abstract

**Purpose:**

The aim of the study was to prospectively evaluate the agreement between chest magnetic resonance imaging (MRI) and computed tomography (CT) and to assess the diagnostic performance of chest MRI relative to that of CT during the follow-up of patients recovered from coronavirus disease 2019.

**Materials and methods:**

Fifty-two patients underwent both follow-up chest CT and MRI scans, evaluated for ground-glass opacities (GGOs), consolidation, interlobular septal thickening, fibrosis, pleural indentation, vessel enlargement, bronchiolar ectasia, and changes compared to prior CT scans. DWI/ADC was evaluated for signal abnormalities suspicious for inflammation. Agreement between CT and MRI was assessed with Cohen’s *k* and weighted *k*. Measures of diagnostic accuracy of MRI were calculated.

**Results:**

The agreement between CT and MRI was almost perfect for consolidation (*k* = 1.00) and change from prior CT (*k* = 0.857); substantial for predominant pattern (*k* = 0.764) and interlobular septal thickening (*k* = 0.734); and poor for GGOs (*k* = 0.339), fibrosis (*k* = 0.224), pleural indentation (*k* = 0.231), and vessel enlargement (*k* = 0.339). Meanwhile, the sensitivity of MRI was high for GGOs (1.00), interlobular septal thickening (1.00), and consolidation (1.00) but poor for fibrotic changes (0.18), pleural indentation (0.23), and vessel enlargement (0.50) and the specificity was overall high. DWI was positive in 46.0% of cases.

**Conclusions:**

The agreement between MRI and CT was overall good. MRI was very sensitive for GGOs, consolidation and interlobular septal thickening and overall specific for most findings. DWI could be a reputable imaging biomarker of inflammatory activity.

## Introduction

To date, no proper and standardized pathway has been established to conduct follow-up after the resolution of patients’ clinical symptoms of coronavirus disease 2019 (COVID-19) and after the real-time polymerase chain reaction (RT-PCR) sampling has turned negative.

To cope with the COVID-19 emergency, it is essential to ensure the prompt discharge of healed patients while at the same time confirming their non-infectivity. COVID-19 patients’ discharge criteria vary in different countries. In Italy, the Superior Health Council of the Ministry of Health stated that a patient hospitalized for COVID-19 can be considered healed after the resolution of symptoms and after two RT-PCR tests for severe acute respiratory syndrome coronavirus 2 (SARS-CoV-2), the virus that causes COVID-19, performed at least 24 h apart are negative. In patients with remission of symptoms within seven days after onset, it is recommended to perform the control test (RT-PCR) at least 7 days after the first test. In China, however, it was deemed necessary to assess the reduction of signs of pulmonary involvement by also conducting a diagnostic imaging examination. Despite the variability in the abovementioned adopted criteria, a chest imaging examination is commonly performed in all COVID-19 patients prior to discharge.

When assessing the natural history of SARS-CoV-2 infection, diagnostic imaging has shown applications for presumptive diagnosis, monitoring, and follow-up of the disease [1–4]. Chest X-ray is commonly performed and can reveal patchy ground-glass opacities (GGOs) and consolidation but is much less sensitive as an imaging modality relative to computed tomography (CT) [5]. Lung ultrasound is a fast bedside examination often completed in the emergency department and intensive care unit [6]. In the setting of COVID-19, it is a frequently performed examination since it is able to show signs of interstitial lung disease (ILD), peripheral consolidation, and acute respiratory distress syndrome [7], although it remains an operator-dependent assessment and it is not considered a sensitive test for the diagnosis of COVID-19. CT has already been demonstrated to be a highly sensitive—albeit not specific—modality for the diagnosis of COVID-19 [7, 8] and CT findings for the disease have been reported to follow a relatively typical temporal pattern and make it possible to monitor the evolution of lung involvement during the clinical course of the condition [9–12].

Recent evidence in the literature showed MRI to be a reliable diagnostic tool in COVID-19 patients. The most common finding in CT was ground-glass opacities in 29 patients (90.6%), followed by consolidation in 14 patients (43.75%) [13]. MRI has been shown to be feasible and a potential alternative to CT and X-ray in several settings, mostly in ILD [14–18]. Specifically, it may take on a promising role in ILD patients for differentiating inflammatory and fibrotic changes. Chest MRI provides different functional information than CT by using diffusion-weighted imaging (DWI) [19].

A codified follow-up algorithm for healed COVID-19 patients does not exist, to our knowledge. The present study therefore sought as primary endpoint to confirm the validation of chest MRI as an imaging tool for the follow-up of COVID-19 patients by correlating its findings with CT scan findings. As a secondary endpoint, the authors investigated the role of the DWI technique in detecting acute inflammation.

## Methods

### Study design

The local ethical committee approved this study and written informed consent for participation in the study was obtained from all enrolled patients. A cohort of 52 patients was prospectively enrolled in the study. Inclusion criteria included prior COVID-19 confirmed by RT-PCR testing, prior CT scan during the active phase of the disease documenting imaging signs compatible with those known to be related to COVID-19, remission of clinical symptoms, negative PCR outcomes of two separate tests performed 24 h apart and an indication to undergo a follow-up CT scan to document the resolution of prior imaging findings. Patients with absolute contraindications to MRI were excluded.

RT-PCR (RealStar® SARS-CoV-2; Altona Diagnostics, Hamburg, Germany) was performed following the RNA extraction (QIAamp® Viral RNA; Qiagen, Hilden, Germany) of samples collected using upper nasopharyngeal as well as oropharyngeal swabs, both to confirm the diagnosis and to document recovery.

### MRI protocol

MRI scans were performed between March 19th and May 12th, 2020 using a 1.5-Tesla magnet (MAGNETOM® Avanto; Siemens Healthcare, Erlangen, Germany) with a 16-channel arrayed surface coil. The imaging protocol included an end-expiratory triggered, proton density (PD)-weighted, fat-saturated sequence acquired on both the axial and coronal planes at a slice thickness of 4 mm and a DWI axial sequence with three different b values of 0, 500, and 1000 and ADC map computation. In case respiratory-triggered images were not of adequate quality due to irregular breathing, a breath-hold echo-planar, fast spin-echo, T2-weighted, fat-saturated axial sequence was alternatively acquired [20]. See Table 1 describing MRI parameters.

**Table 1:** MRI acquisition parameters for PDW and DWI

1.5 T scan	PDW	DWI
TE	27	76
TR	1265	11,900
Flip angle	150	NA
Matrix	256 × 192	125X192
Bandwidth	592	1736
FOV	400 mm	360 mm
Slice thickness	4 mm	5 mm
No. of echo	1	1

PDW, Proton Density Weighted; DWI, Diffusion-Weighted Imaging; TE, Echo Time; TR, Repetition time; FOV, Field of View

### CT protocol

The routine CT protocol consisted of volumetric end-inspiratory and end-expiratory low-dose scans acquired using either 16- and 64-rows scanners (Somatom Sensation 16 and Somatom Sensation 64; Siemens Healthcare, Erlangen, Germany), or a 64-row multi-detector scanner (Somatom Definition; Siemens Medical Solutions, Forehheimen, Germany). Images were acquired in the end-inspiratory phase with the patient in a supine position. Scanning parameters were as follows: tube voltage, 120 kVp; tube current, 100–250 mAs; pitch, 1.2; and collimation, 0.625–0.75 mm. Images were reconstructed using a 1-mm slice thickness on axial and coronal planes, using both soft tissue kernel (B31f) and lung kernel (B75f) reconstruction.

### Image analysis

CT and MRI images were reviewed independently by two radiologists, both with more than 10 years of experience in body imaging and MRI. Patients were randomly split into two groups (Group A and Group B); then, one radiologist (*BLINDEND*) analyzed the MRI scans of patients in Group A and CT images of patients in Group B, while the second radiologist (*BLINDED*) analyzed the MRI scans of patients in Group B and CT images of patients in Group A. Each radiologist had access only to one follow-up scan (either CT or MRI), while both of them could examine the prior CT scan performed during the period of active disease, to make comparisons and assess for changes. For each follow-up scan, radiologists examined the following lung parenchymal imaging findings: GGOs, consolidation, interlobular septal thickening, fibrosis, pleural indentation, vessel enlargement, and bronchiolar ectasia. For each imaging finding, a binary score indicating its presence or absence was assigned on both CT and MRI scans. An overall score of one to three points was assigned to each scan, indicating the degree of change from the previous CT scan, where a score of one point indicated an improvement, a score of two points indicated stability, and a score of three points indicated a worsened status. For MRI only, DWI images were qualitatively assessed and assigned a binary score, indicating a low or high probability of inflammatory activity, based on the presence of hyperintensity on both DWI and on ADC maps. Radiological terms such as GGO, pulmonary consolidation, interlobular septal thickening, and bronchiolectasis were used according to the Fleischner Society's glossary for thoracic imaging [21]. Vessel enlargement, indicated also as vascular thickening, vascular enhancement, “micro-vascular dilatation sign,” bronchovascular enlargement or “dandelion fruit sign,” is generally defined as a thickened aspect on chest CT of blood vessels which flow through or by GGOs [22].

### Statistical analysis

Concordance between CT (standard of reference) and MRI scans in the assessment of the presence or absence of different imaging findings was calculated using the Cohen’s *k* statistic. Meanwhile, the weighted Cohen’s *k* statistic was used to assess for concordance in the quantitative score indicating the overall change that had occurred from the previous CT scan. The degree of agreement based on these *k* values was interpreted as follows: below 0.4, poor agreement; between 0.41 and 0.60, moderate agreement; between 0.61 and 0.80, substantial agreement; and between 0.81 and 1, almost perfect agreement.

The sensitivity, specificity, accuracy, positive predictive values, and negative predictive values of MRI for the detection of each imaging findings were calculated, considering CT as the reference standard.

Statistical analysis was performed with the software package R (version 3.6.3; R Foundation for Statistical Computing, Vienna, Austria).

## Results

Fifty-two patients [31 males and 21 females, mean age: 58 years, interquartile range (IQR): 53–66 years] were enrolled in the study. All patients were asymptomatic at the time of follow-up imaging and underwent MRI and CT scans within ± one day. Imaging exams were performed at an average of 11 days (IQR: 11.25–23 days) after complete clinical remission, confirmed by two consecutive negative results of RT-PCR tests for SARS-CoV-2. The room time for CT imaging was 4.3 min (IQR 3.7–5.3 min), the dose length product was 104 mGy × cm (IQR: 90–113 mGy × cm), and the effective dose was 1.3 mSv (IQR: 0.9–1.8 mSv). Separately, the MRI room time was 19 min (IQR 12–23 min). MRI images were technically adequate and of appropriate diagnostic quality in all cases. In 51 of 52 cases (98.1%), the PD-weighted, fat-saturated turbo spin-echo sequence with respiratory triggering was acquired. In one of 52 cases, a breath-hold echo-planar, fast spin-echo, T2-weighted protocol was used instead of the PD-weighted sequence due to the patient’s irregular breathing. DWI was technically adequate in 50 of 52 cases (96.1%), while, in two of 52 cases (3.8%), the presence of artifacts impeded the appropriate evaluation of DWI.

**Fig. 1:**
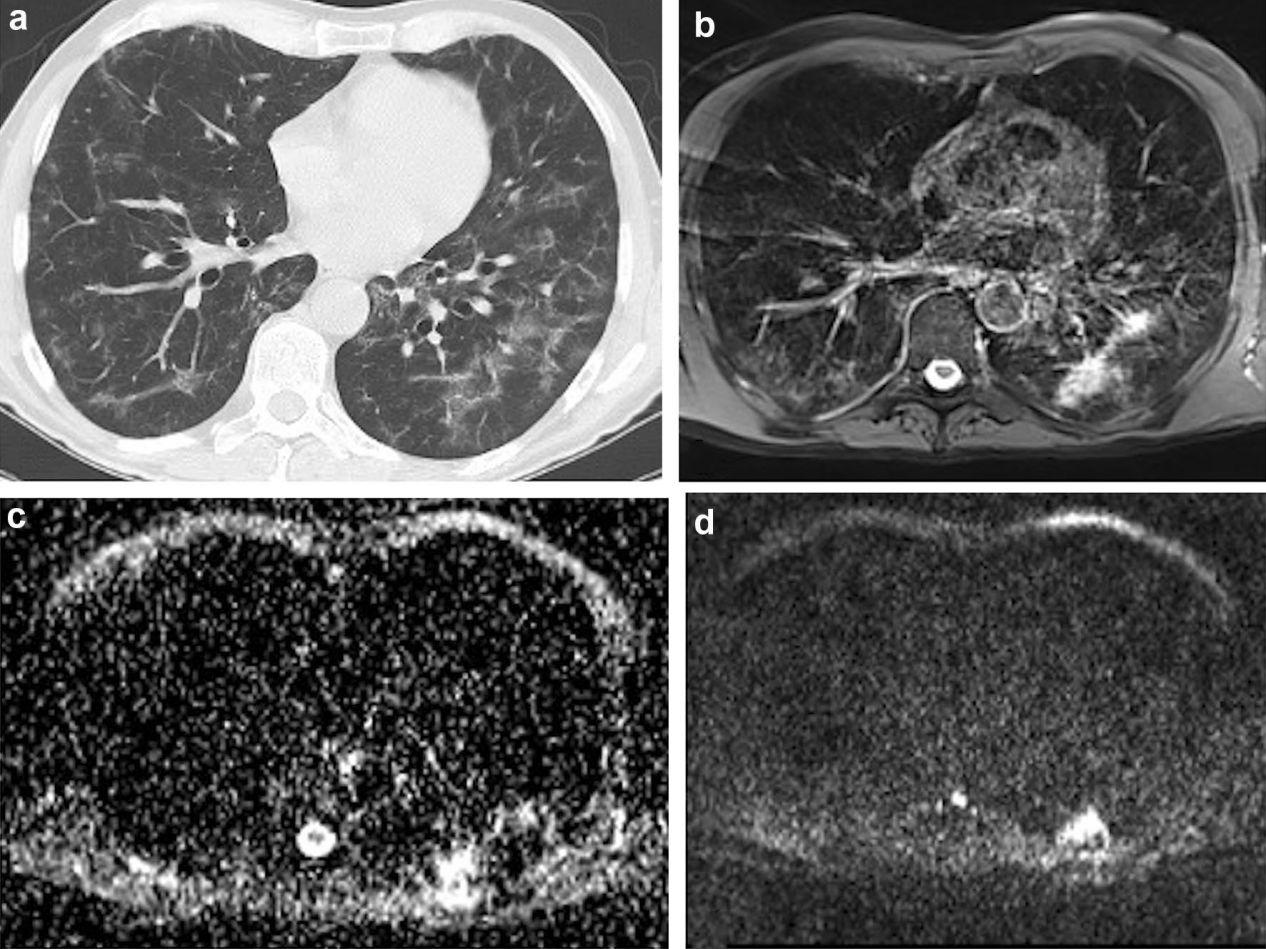
Ground-glass opacities with positive DWI. 58-year-old male. **a** End-inspiratory axial CT, **b** Free-breathing PD-weighted axial image, **c** Apparent Diffusion Coefficient Map, and d) DWI free breathing (*b* = 1000 s/mm^2^). Note an area of GGO on the left lower lobe that shows restricted diffusion on DWI and hyperintensity on ADC map, corresponding to an area of acute inflammation

The imaging findings assessed on MRI and CT scans for this study are summarized in Table 2. The predominant patterns of parenchymal involvement according to CT versus MRI were: GGOs in 27 of 52 cases (51.9%) vs. 32 of 52 cases (61.5%), interlobular septal thickening in 17 of 52 cases (32.7%) vs. 17 of 52 cases (32.7%), and consolidation in two of 52 cases (3.8%) vs. two of 52 cases (3.8%). At least one additional finding was present in 41 of 52 cases (78.8%) according to CT and in 47 of 52 cases (90.4%) according to MRI. No pulmonary findings were observed in five of 52 cases (9.6%) using CT and in one of 52 cases (1.9%) using MRI. DWI was positive for high suspicion of inflammatory activity in 23 to 50 cases (46.0%); 19 of whom also had GGOs (Fig. 1). Agreement between CT and MRI when evaluating the presence of different imaging findings, as assessed with Cohen’s *k,* was very high for consolidation (*k* = 1.000); substantial for interlobular septal thickening (*k* = 0.734); poor for GGOs (*k* = 0.339), fibrotic changes (*k* = 0.224), pleural indentation (*k* = 0.231), vessel enlargement (*k* = 0.339), and bronchiolar ectasia (*k* = 0.000). The degree of agreement between CT and MRI in identifying the predominant pattern was substantial (*k* = 0.764).

**Table 2:** Prevalence of imaging findings assessed on CT and MRI

	No. of patients with imaging finding	
	CT	MRI
GGO	40/52 (76.9%)	49/52 (94.2%)
Consolidation	8/52 (15.4%)	8/52 (15.4%)
Fibrosis	17/52 (32.7%)	3/52 (5.8%)
Interlobular septal thickening	41/52 (78.8%)	45/52 (86.5%)
Pleural indentation	26/52 (50.0%)	6/52 (11.5%)
Vessel enlargement	4/52 (7.7%)	6/52 (11.5%)
Bronchiolar ectasia	19/52 (36.5%)	0/52 (0.0%)
DWI	N/A	23/50 (46.0%)

GGO, Ground-Glass Opacity; DWI, Diffusion-Weighted Imaging

Meanwhile, the level of agreement between CT and MRI when assessing the change from the previous CT scan was almost perfect, with a weighted Cohen’s *k* value of 0.857.

**Fig. 2:**
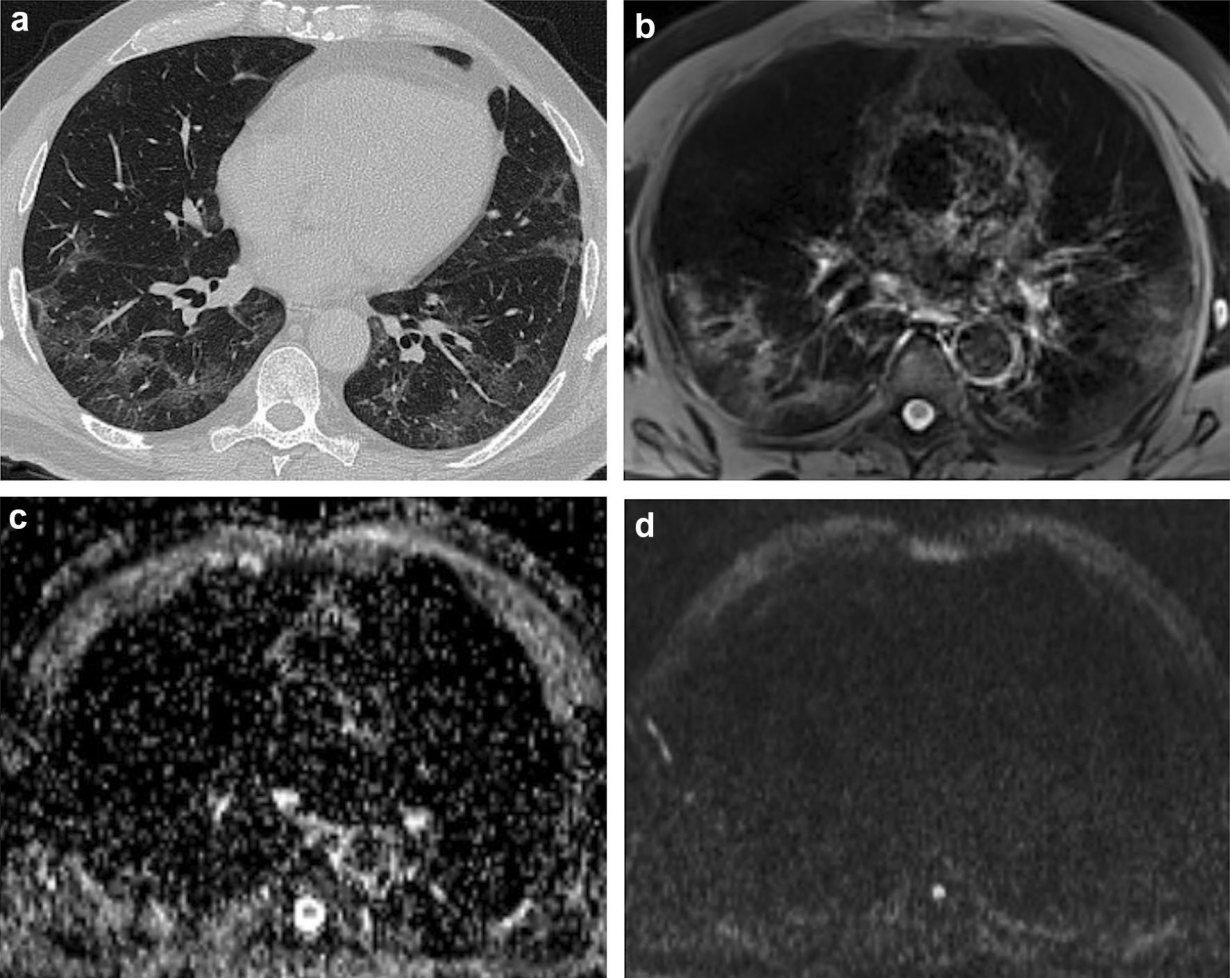
Interlobular septal thickening on ground-glass opacity. 56-year-old male. **a** End-inspiratory axial CT after 1 month from discharge, **b** Free-breathing PD-weighted axial image after one month from discharge, **c** Apparent Diffusion Coefficient Map, and **﻿d** diffusion-weighted imaging (DWI) free breathing (*b* = 1000 s/mm^2^). Note areas of interlobular septal thickening superimposed to a GGO, on both the right and the left lower lobes that do not show restricted diffusion on DWI nor hyperintensity on ADC map

The sensitivity, specificity, accuracy, and positive and negative predictive values of MRI, when considering CT as the standard of reference, in the detection of different parenchymal patterns are detailed in Table 3. The sensitivity was highest for interlobular septal thickening (1.00, 95% confidence interval (CI): 0.91–1.00), consolidation (1.00, 95% CI: 0.63–1.00), and GGOs (1.00, 95% CI: 0.91–1.00) (Fig. 2). Conversely, MRI showed poor sensitivity in the detection of fibrosis (0.18, 95% CI: 0.04–0.43), pleural indentation (0.23, 95% CI: 0.09–0.44), vessel enlargement (0.50, 95% CI: 0.07–0.93), and bronchiolar ectasia (0.00, 95% CI: 0.00–0.18). Specificity was overall very high, except for GGOs (0.25, 95% CI: 0.05–0.57) and interlobular septal thickening (0.64, 95% CI: 0.31–0.89). MRI was not able to detect bronchiolar ectasia in any of the cases.

**Table 3:** Chest MRI and CT Agreement and MRI Diagnostic Performance to assess different alteration pattern

	Agreement	Sensitivity	Specificity	Accuracy	NPV	PPV
GGO	0.339	1.00 (0.91, 1.00)	0.25 (0.05, 0.57)	0.83 (0.70, 0.92)	1.00 (0.29, 1.00)	0.82 (0.68, 0.91)
Consolidation	1.000	1.00 (0.63, 1.00)	1.00 (0.92, 1.00)	1.00 (0.93, 1.00)	1.00 (0.92, 1.00)	1.00 (0.63, 1.00)
Fibrosis	0.224	0.18 (0.04, 0.43)	1.00 (0.90, 1.00)	0.73 (0.59, 0.84)	0.71 (0.57, 0.83)	1.00 (0.29, 1.00)
Interlobular septal Thickening	0.734	1.00 (0.91, 1.00)	0.64 (0.31, 0.89)	0.92 (0.81, 0.98)	1.00 (0.59, 1.00)	0.91 (0.79, 0.98)
Pleural indentation	0.231	0.23 (0.09, 0.44)	1.00 (0.87, 1.00)	0.61 (0.47, 0.75)	0.57 (0.41, 0.71)	1.00 (0.54, 1.00)
Vessel enlargement	0.339	0.50 (0.07, 0.93)	0.92 (0.80, 0.98)	0.88 (0.76, 0.96)	0.96 (0.85, 0.99)	0.33 (0.04, 0.78)
Bronchiolar ectasia	0.000	0.00 (0.00, 0.18)	1.00 (0.89, 1.00)	0.63 (0.49, 0.76)	0.63 (0.63, 0.63)	N/A
Predominant pattern	0.764	N/A	N/A	0.86 (0.74, 0.94)	N/A	N/A
Change from prior CT	0.857	N/A	N/A	0.94 (0.84, 0.99)	N/A	N/A

NPV, Negative Predictive Value; PPV, Positive Predictive Value; GGO, Ground-Glass Opacity

## Discussion

To our knowledge, the role of chest MRI in COVID-19 patients has not been investigated to date. In light of this, the present study revealed the promising results regarding MRI’s reliability and appropriateness in the evaluation of COVID-19 patients’ follow-up as compared with CT as the standard imaging modality.

**Fig. 3:**
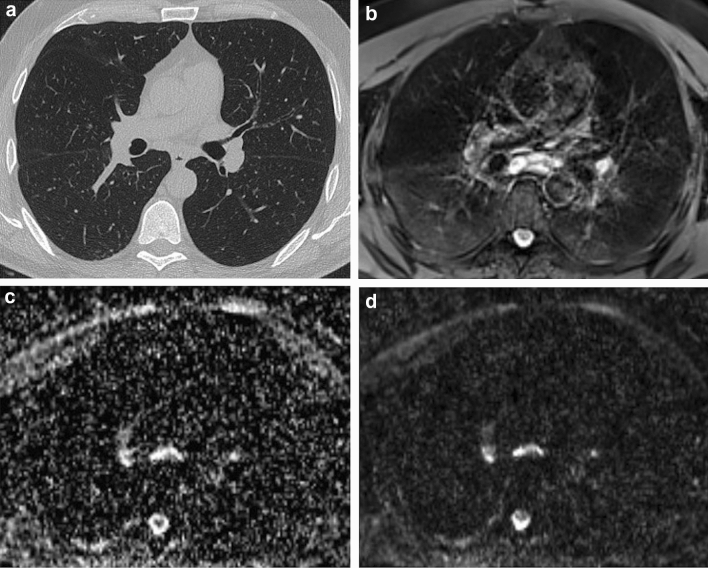
Ground-glass opacity with negative CT. 46-year-old male. **a** End-inspiratory axial CT after 1 month from discharge, **b** Free-breathing PD-weighted axial image, **c** Apparent Diffusion Coefficient Map, and **d** diffusion-weighted imaging (DWI) free breathing (*b* = 1000 s/mm^2^). Note areas of GGO on both the right and the left lower lobes visible on the PD-weighted image not corresponding to any alteration on the CT scan. No restricted diffusion on DWI nor hyperintensity on ADC map depicted

The main disease pattern, among GGOs, consolidation, interlobular septal thickening, and fibrosis, was detected by both MRI and CT with substantial agreement (*k* = 0.764). Also, MRI proved to be very reliable in assessing the resolution or progression of radiological signs relative to the previous CT scan, showing almost perfect agreement (*k* = 0.857) with CT. Of note, very good agreement was confirmed for consolidation and interlobular septal thickening (*k* = 1.00 and *k* = 0.734, respectively). However, poor agreement was noted concerning the detection of GGOs (*k* = 0.339), which pathologically corresponds to partial filling of the alveolar lumen with fluid, macrophages, neutrophils, or amorphous material and which correlates with disease activity [23]. The poor agreement between MRI and CT in the detection of GGOs could be explained by MRI’s greater ability to discriminate the alveolar content by quantifying its PD, showing a relatively much higher signal in lung parenchyma affected by endo-alveolar effusion when compared with normally aerated lungs (Fig. 3). Accordingly, the sensitivity for MRI in detecting GGOs was perfect (1.00, 95% CI: 0.91–1.00) as compared with the very low specificity (0.25, 95% CI: 0.05–0.57). Separately, it is noteworthy that very poor agreement was recorded for the detection of bronchiolar ectasia (*k* = 0.000). These data confirm previous findings by Ciet et al. [24], who reported the superior sensitivity of CT when evaluating changes in the peripheral areas of the lung, such as bronchiolar ectasia. A possible explanation for this could be the lower spatial resolution of MRI as compared with CT, especially when focusing on more distal areas of the lung [25]. To overcome the issue, three-dimensional T1-weighted, gradient-echo sequences might be added to the protocol. However, further studies are necessary to confirm these radiologic and pathologic correlations.

The agreement between chest MRI and CT imaging has previously been evaluated in the assessment of different pathologies, such as diffuse pulmonary damage and cystic fibrosis [19, 26–30]. In 2015, Milito et al. showed that MRI with DWI was a reliable technique for detecting lung alterations in patients with primary antibody deficiencies. In 2016, Tepper et al. investigated the role of MRI during the follow-up of pediatric patients with cystic fibrosis, demonstrating that the association of MRI scan findings and clinical parameters validates chest MRI as a method for monitoring lung disease in this patient population [30].

Considering our secondary aim, 46% of study participants showed a high degree of signal intensity on both DWI and the ADC map, suggesting the possibility that alveolar infiltrates cause acute inflammation in this context. The role of DWI MRI has previously been validated and proposed for the detection of pulmonary inflammation [19, 27, 28]. In 2017, Ciet et al. [29] showed how DWI can detect acute inflammatory reactions during respiratory tract exacerbations in patients with cystic fibrosis.

Such findings might have important clinical outcomes in terms of the selection of candidates to undergo prolonged anti-inflammatory treatment despite confirmation of viral clearance. However, comparisons with clinical and laboratory data are of paramount importance in future clinical studies to validate the role of DWI in this clinical setting. The association of hyperintense *foci* on DWI and ADC maps and the presence of inflammatory infiltrates are supported by the publication of recent pathologic series of postmortem lung biopsies in COVID-19 patients, in which, among others, lymphocytes, multi-nucleated giant cells, large atypical pneumocytes, and no viral particles were detected [31–33]. Our results confirm what has been suggested by Torkian et al. [34], who recently showed that chest MRI could be a valuable tool for the follow-up of targeted at-risk COVID-19 patients. Of note, one of the possible obstacles to including chest MRI in COVID-19 follow-up protocols at institutions with limited resources is the potential work overload of MRI systems and the need to dedicate staff members to this initiative. However, in large centers currently committed to the management of COVID-19, there has been a large decrease in nonurgent/routine outpatient MRI requests, and it may be feasible to establish a dedicated MRI room, as has been done at our institution. More generally, the main limitations of this study that should be addressed include the relatively small number of patients included, a lack of expertise that might limit the reproducibility of the proposed method and the need to dedicate an MRI room to be able to deal with the needs of both nonurgent non–COVID-19 and COVID-19–associated cases. Further prospective studies investigating MRI performance as compared with clinical and laboratory data among larger cohorts of patients are also needed to validate the role of chest MRI in the follow-up of COVID-19.

The use of chest MRI has been increasing in recent years, since there is no radiation risk. MRI has been demonstrated to be a reliable tool in the follow-up of COVID-19 patients—being, notably, a radiation-free, minimally invasive imaging modality—after clinical signs and symptoms have resolved and negative PCR test results are confirmed. DWI MRI might constitute a promising imaging biomarker in the evaluation of a pulmonary active inflammatory process, which might influence decision-making in the patient’s clinical management course, and the long-term impact on lung health status.

## Data Availability

The data that support the findings of this study are available from the corresponding author, (VP), upon reasonable request.
